# *Bacillus* *megaterium* DSM 32963 Enhances Specialized Pro-Resolving Mediator Production from an *n*-3 PUFA Salt in a Dynamic Model of the Human Intestine

**DOI:** 10.3390/metabo15020105

**Published:** 2025-02-07

**Authors:** Bodo Speckmann, Paul M. Jordan, Oliver Werz, Robert K. Hofstetter, Ellen Ehring, Marie-Luise Vogel, Koen Venema

**Affiliations:** 1 Evonik Operations GmbH, Rodenbacher Chaussee 4, 63457 Hanau, Germany; ellen.ehring@evonik.com (E.E.); marie-luise.vogel@evonik.com (M.-L.V.); 2 Department of Pharmaceutical/Medicinal Chemistry, Institute of Pharmacy, Friedrich Schiller University Jena, 07743 Jena, Germany; paul.jordan@uni-jena.de (P.M.J.); oliver.werz@uni-jena.de (O.W.); robert.klaus.hofstetter@uni-jena.de (R.K.H.); 3 Jena Center for Soft Matter (JCSM), Friedrich Schiller University Jena, 07743 Jena, Germany; 4 Centre for Healthy Eating & Food Innovation, Campus Venlo, Maastricht University, Villafloraweg 1, 5928 SZ Venlo, The Netherlands; koen.venema@outlook.com

**Keywords:** omega-3, probiotic, metabiotic, synbiotic, inflammation resolution, nutritional therapy, inflammatory bowel diseases, irritable bowel syndrome

## Abstract

Background: Omega-3 polyunsaturated fatty acids (*n*-3 PUFA) have been used in the treatment of inflammatory bowel diseases (IBD) and irritable bowel syndrome (IBS), and their effects are potentiated upon conversion to specialized pro-resolving mediators (SPM). Recent studies indicated that the probiotic bacterial strain *Bacillus megaterium* DSM 32963 can be used to enhance the production of SPM and its precursors in vivo. Methods: Here, we explored the contribution of *Bacillus megaterium* DSM 32963 to SPM production in a validated, dynamic model of the upper and lower intestine. The TIM-1 and TIM-2 models were applied, with the TIM-2 model inoculated with the fecal microbiota of healthy individuals and probed with an *n*-3 PUFA lysine salt with and without *Bacillus megaterium* DSM 32963 or an SPM-enriched fish oil or placebo. Kinetics of SPM production were assessed by metabololipidomics analysis, and survival and engraftment of the *Bacillus megaterium* strain was monitored by plate counting and by strain-specific qPCR. Results: *Bacillus megaterium* DSM 32963 poorly survived TIM-1 conditions but propagated in the TIM-2 model, where it enabled the metabolism of *n*-3 PUFA to SPM (resolvin E2 and protectin DX) and SPM precursors (e.g., 5-hydroxyeicosapentaenoic acid (5-HEPE), 15-HEPE, 18-HEPE, 4-hydroxydocosahexaenoic acid (4-HDHA), 10-HDHA, and 17-HDHA, among other EPA- and DHA-derived metabolites) with significantly higher levels of lipid mediator production compared to the *n*-3 PUFA lysine salt alone; esterified *n*-3 PUFA were hardly converted by the microbiota. Conclusions: These findings reinforce that *Bacillus megaterium* DSM 32963 facilitates SPM production in situ from bioavailable *n*-3 PUFA in the large intestine, highlighting its use to complement eukaryotic SPM biosynthesis by the host and its possible therapeutic use for, e.g., IBD and IBS.

## 1. Introduction

Gut microbial metabolites are crucial mediators of the microbiota–host health relationship [1,2,3]. Their formation is shaped by three main factors: the availability of substrates (dietary components, drugs, xenobiotics, etc.), the composition/activity of the microbiota, and host functions (control of gastrointestinal motility, absorption, excretion, digestion, etc.). Importantly, the gut microbial metabolome can be expanded and tailored through microbiome-targeting or synbiotic strategies. A recently described concept of synbiotics aims for the production of defined and health-beneficial metabolites from substrates through microbes with specific metabolic functions (so-called metabiotics) [4]. In that way, targetable metabolites include, for example, spermidine [5], urolithin A [6], indole derivatives [7], and lipid mediators (LM) [8]. A prominent class of LM is specialized pro-resolving mediators (SPM), comprising resolvins, maresins, protectins, and lipoxins, which are formed by host enzymes from *n*-3 PUFA, such as eicosapentaenoic acid (EPA) and docosahexaenoic acid (DHA), and trigger inflammation resolution [9,10]. SPMs are a promising class of substances for the prevention and treatment of several diseases that are triggered by chronic inflammation [10,11]. On the other hand, their formation and/or subsequent signaling events can be impaired, as indicated for IBD [12,13,14,15], and, therefore, strategies to increase endogenous SPM formation/levels may be advantageous. We pursued a synbiotic strategy for the self-sufficient microbial biosynthesis of SPM and its precursors in vivo to provide the body with these metabolites independently from the eukaryotic metabolic capacity of the host. To this end, we recently discovered that the species *Bacillus megaterium* produces a spectrum of SPM and precursors in single-strain cultivations [8]. *Bacillus megaterium* is a common soil bacterium found also in various other habitats, including gastrointestinal tracts, where it is one of the fewer oxygen-consuming taxa. It is unique among other (gut) microorganisms, as it expresses CYP102A1, also named CYP450BM-3, a bifunctional enzyme that catalyzes the NADPH-dependent hydroxylation of numerous substrates, e.g., *n*-3 PUFA, via consecutive oxygenase and reductase activities [16,17]. We reported extensive variations in the amino acid sequence of CYP450BM-3 in Bacillus megaterium strains, which also showed variability in the composition and amounts of EPA- and DHA-derived SPM [8]. These reactions were dependent on the availability of *n*-3 PUFA as free fatty acids (FFA). Further, a synbiotic composition comprising *Bacillus megaterium* DSM 32963 and an *n*-3 PUFA FFA lysine salt (SynΩ3) significantly increased plasma levels of several EPA- and DHA-derived pro-resolving LM in humans, also, in comparison to a fish oil control with similar *n*-3 PUFA content [18].

These results argued for an intraluminal biosynthesis of LM through SynΩ3 followed by their absorption into the circulation. We hypothesized that *Bacillus megaterium* DSM 32963 can thrive under gastrointestinal conditions, especially in the colon, where it produces relevant amounts of SPM and SPM precursors from *n*-3 PUFA provided in the form of FFA lysine salts. The present study was performed to test this hypothesis by assessing the behavior of SynΩ3 under simulated conditions of the gastrointestinal tract and in comparison to treatments with *n*-3 PUFA alone. The TNO in vitro models of the gastrointestinal tract (nicknamed TIM) simulate to a high degree the successive dynamic processes in the stomach and small intestine (TIM-1) and in the large intestine (TIM-2) and are predictive for what happens in human individuals [19,20]. The TIM-1 and TIM-2 systems were applied and probed with SynΩ3, *n*-3 PUFA lysine salt (i.e., SynΩ3 without *Bacillus megaterium* DSM 32963), SPM-precursor-enriched fish oil, and control. Based on the results, we conclude that *n*-3 PUFA as FFA—but not in their esterified form—can be converted by a large intestinal microbiota to several LM and that this conversion is strongly enhanced by the presence of *Bacillus megaterium* DSM 32963.

## 2. Materials and Methods

### 2.1. Test Samples

The TIM-1 and TIM-2 experiments were performed with the following three different *n*-3 PUFA compositions and under control conditions (=nothing added).

### 2.2. SynΩ3

SynΩ3 is the content of one capsule of the dietary supplement (IN VIVO BIOTICS™ resolving, Evonik Operations GmbH, Hanau, Germany). One capsule contains ≥1 billion colony-forming units of *Bacillus megaterium* DSM 32963 (B4U™63) and *n*-3 PUFA lysine salt equivalent to 83.3 mg EPA and 41.7 mg DHA (AvailOm^®^, Theodore, AL, USA). The capsules were coated with a pH-dependent release formulation (EUDRAGUARD^®^biotic functional coating, Theodore, AL, USA) for targeted delivery of the ingredients into the large intestine.

### 2.3. Ω3 Salt (n-3 PUFA Lysine Salt)

In total, 30 mg *n*-3 PUFA lysine salt (AvailOm^®^, Evonik) contains 83.3 mg EPA and 41.7 mg DHA.

### 2.4. Fish Oil (SPM-Precursor-Enriched Fish Oil)

One capsule of the dietary supplement SPM Active^®^ (Metagenics, Ostend, Belgium) contains 500 mg fish oil concentrate, equivalent to 54 mg EPA and 108 mg DHA, as well as ~100 µg 18-HEPE, 32 µg 17-HDHA, and 76 µg 14-HDHA [21].

### 2.5. Set-Up of TIM-1

**Figure 1:**
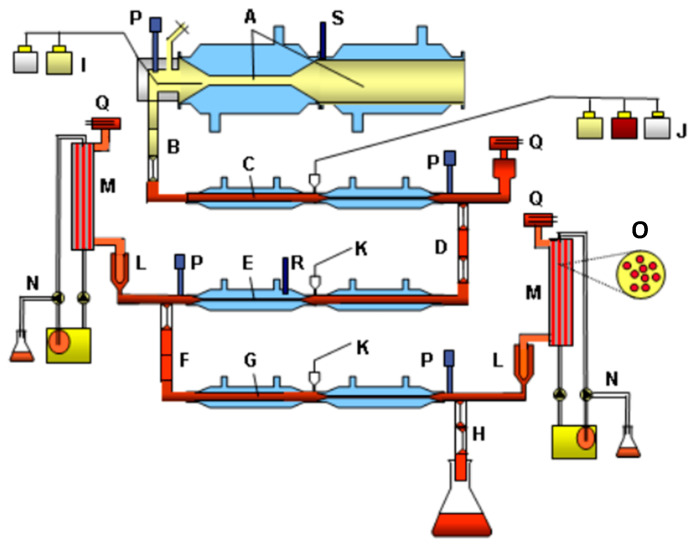
Schematic diagram of the dynamic, multi-compartmental TNO in vitro model of the stomach and small intestine (TIM-1). (A) Stomach compartment; (B) pyloric sphincter; (C) duodenum compartment; (D) peristaltic valve; (E) jejunum compartment; (F) peristaltic valve; (G) ileum compartment; (H) ileo-cecal sphincter; (I) stomach secretion; (J) duodenum secretion; (K) jejunum/ileum secretion; (L) pre-filter; (M) semi-permeable membrane; (N) water absorption; (O) hollow fiber system (cross-section); (P) pH electrodes; (Q) level sensors; (R) temperature sensor; (S) pressure sensor. Figure taken from https://doi.org/10.1016/j.ejps.2013.08.024 (accessed on 20 January 2025).

In the model comprising the stomach and small intestine (TIM-1; see Figure 1 for schematic), the experiments were performed under the average physiological conditions as found in the GI tract for human adults, as described before [22], with the exception that filtration membranes were used, instead of dialysis membranes, to remove mixed micelles containing lipids and SPMs, simulating absorption by the body. The gastric emptying, intestinal residence time, and gastric pH curve and pH in the small-intestinal compartments mimicked the situation as found in human adults. Moreover, the concentrations of electrolytes, enzymes, bile, bile salts, and pancreatic juice were adjusted at the average concentrations as described for adults. The “meal” with the test samples was introduced into the gastric compartment containing 5 mL gastric residue at pH 2. For each experiment, the secretion products (e.g., gastric juice with enzymes, electrolytes, dialysis liquids, bile, and pancreatin) were freshly prepared, the pH electrodes calibrated, and the filtration membranes (hollow fiber units) replaced. The starting pH of the meal was set at 6.5, simulating intake of the capsules with an actual meal, like breakfast. Since *B. megaterium* was present and added as spores, LB medium was used in these experiments as meal, since it contains triggers for the spores to germinate, which was required to induce conversion of EPA to SPMs. Also, the blank contained LB medium (Table 1).

The gastric contents passed the pyloric valve into the duodenal compartment according to the computer-controlled gastric emptying curve. In the duodenal compartment, the gastric content was neutralized to pH 6.5 ± 0.2, and bile and pancreatin were secreted. The contents of the duodenum (passage time ± 10 min) were delivered into the jejunum compartment (pH 6.8 ± 0.2) and, after that, into the ileum compartment (pH 7.2 ± 0.2). In each compartment, the physiological concentrations of bile salts, pancreatic enzymes, and electrolytes were simulated in combination with an average physiological passage of the chyme through the small intestine. Filtration units were connected to the jejunum and ileum, which absorbed digestion products (such as FFAs) and produced SPMs and water (Figure 1M). At the end of the ileum compartment (Figure 1H), the simulated “ileo-cecal valve” delivered the intestinal contents into a sampling bottle at 0–4 °C. Every hour for a total of 6 h, the collected volume was measured, and representative subsamples were analyzed for SPMs as described below. The concentrations in the samples were used to determine the cumulative bioaccessibility.

### 2.6. Set-Up of TIM-2

**Figure 2:**
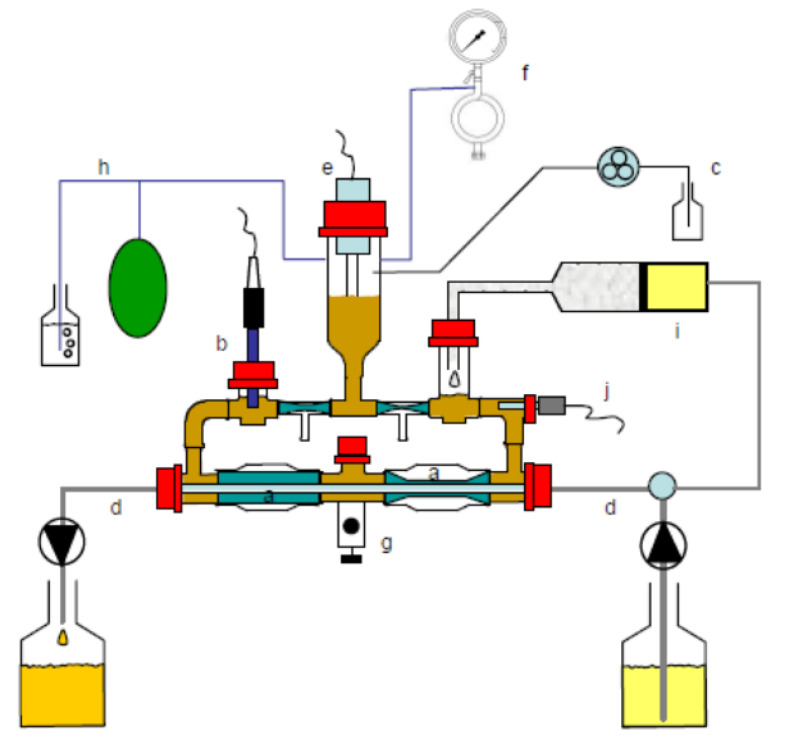
Schematic diagram of the dynamic, multi-compartmental TNO in vitro model of the colon (TIM-2). (a): Peristaltic compartments; (b): pH electrode; (c): alkali pump; (d): dialysis liquid circuit with hollow fibers; (e): level sensor; (f): N2 gas inlet; (g): sampling port; (h): gas outlet; (i): “ileal delivery” container; (j): temperature sensor. Image taken from: https://doi.org/10.1016/j.foodchem.2024.141141 (accessed on 20 January 2025).

In the model comprising the large intestinal (colon) compartments (TIM 2; Figure 2), the following standardized conditions were simulated: body temperature (), pH in the lumen (), composition and rate of secretion, delivery of a predigested substrate from the “ileum” (Standard Ileal Efflux Medium; SIEM), mixing and transport of the intestinal contents (), absorption of water and microbial metabolites (), and presence of a complex, high-density, metabolically active, anaerobic microbiota of human adult origin. The microbiota was pooled according to Aguirre et al. [23] to allow for a standardized microbiota that could be used for all experiments. The pool was composed of fecal samples collected from 8 donors (4M:4F, age range 24–53). The model was set up and inoculated with the microbiota on the first day. The microbiota was subsequently fed the SIEM medium during an overnight adaptation period. Thereafter, the test samples were added daily—twice the amount as for the TIM-1—through the feed syringe. Samples were taken every 24 h for a period of 72 h.

### 2.7. Quantification of Bacillus sp. CFU

To determine survival of the added *Bacillus megaterium* DSM 32963 strain in the upper GI tract, samples were collected hourly at the end of TIM-1 and plated on Glucose Yeast Extract BC agar (per liter: peptone 5 g, yeast extract 5 g, glucose 5 g, di-potassium hydrogen phosphate 0.5 g, potassium dihydrogen phosphate 0.5 g, magnesium sulphate 0.3 g, trace mineral solution 1.0 mL, and agar 15 g, pH 6.2; trace mineral solution (per ml): sodium chloride 10 mg, iron (II) sulphate 7H_2_O 18 mg, manganese (II) sulphate H_2_O 16 mg, zinc sulphate 7H_2_O 1.6 mg, copper (II) sulphate 5H_2_O 1.6 mg, and cobalt (II) sulphate 7H_2_O 1.6 mg) directly and after heating at 70 °C for 30 min (to kill vegetative cells). For each sample, a 10-fold dilution series was made in sterile peptone diluent (0.1% peptone in water) and then plated directly or after heat treatment (30 min at 70 °C, then cooling on ice for 5 min). Plates were incubated aerobically at 40 °C for 2 days. The difference in number of colonies between direct plating (total counts) and after heating (spores only) signifies the germinated cells.

In TIM-2, samples were taken from both the lumen and the dialysate at time-point zero (T0, just prior to the addition of the test samples) and after 24 (T24), 48 (T48), and 72 (T72) hrs to study the production of SPMs. Moreover, samples were taken from the lumen for determination of *Bacillus* counts, both vegetative cells as well as spores, using plating on chromogenic *Bacillus* agar plates with clindamycin and polymyxin B (per liter: peptone 10 g, meat extract 1 g, D-mannitol 10 g, sodium chloride 10 g, 5-bromo-4-chloro-3-indolyl-ß-glucopyranoside chromogenic mixture 3.2 g, phenol red 25 mg, agar 15 g, polymyxin B 10 mg, and clindamycin 2 mg; pH 7.1), on which the strain formed yellow, mucoid colonies. Furthermore, a species-specific q-PCR was used to specifically determine the added *B. megaterium* strain (see below).

### 2.8. Quantification of Bacillus megaterium DSM 32963 by qPCR

For strain-specific qPCR-based determination of *Bacillus megaterium* DSM 32963 counts in different TIM-1 and TIM-2 samples, DNA was isolated out of the samples. For mechanical and chemical disruption of the cells, 250 µL of sample was added with 750 µL of lysis buffer ScreenFloX^®^ KF Kit (Evonik Operations GmbH, Wesseling, Germany) to Lysing Matrix B tubes, 2 mL (MP Biomedicals, Santa Ana, CA, USA). As an internal control for DNA isolation and later amplification, 4 µL of Primer Design IC Mix (Internal DNA extraction control, Primer Design, Eastleigh, UK) was added. An additional heat treatment of 20 min at 85 °C with two cycles of mechanical disruption (vibration mill, 10 min, 20Hz) promoted disruption of spores efficiently. DNA isolation was further performed according to ScreenFloX^®^ KF Kit (Evonik Operations GmbH) as recommended. For DNA amplification, a set of specific primers and probe, selected as a unique target for molecular identification and enabling the enumeration of *Bacillus megaterium* DSM 32963 in the DNA eluate, were used (DSM 32963_for TGGTGAAGCATGTAGTAG, DSM 32963_rev TGACTCAACCTTACTCTC, and DSM 32963_probeCGTTCCAAATGGTGGTGT). qPCR was performed for each sample, including internal standards for *Bacillus megaterium* DSM 32963 and negative control, with final concentrations as follows: one-fold Luna Universal Probe qPCR Master Mix (NEB, UK), 400 nM specific primer forward and reverse each, 100 nM specific probe, and 5µl of DNA eluate. DNA amplification was executed using CFX reader (Bio-Rad Laboratories, Inc., Hercules, CA, USA), applying the following 2-step PCR protocol: 95 °C for 10 min, (95 °C for 15 s and 52 °C for 30 s) ×40. During qPCR, the target gene and an internal DNA extraction control were simultaneously amplified and detected by fluorescent dye-labeled probes. The application of quantification standards with known concentrations of the *Bacillus megaterium* ID gene in each qPCR run allows the exact amount of *Bacillus megaterium* target in the sample to be determined.

### 2.9. Analysis of Lipid Mediators in TIM-1 and TIM-2 Samples

Lipid mediator (LM) analysis using ultra-performance liquid chromatography–tandem mass spectrometry (UPLC-MS/MS) was performed as described previously [24], with some minor modifications. Briefly, samples were first mixed with the same volume of ice-cold methanol containing deuterium-labeled internal standards (200 nM *d*8–5*S*-HETE, *d*4-LTB_4_, *d*5-LXA_4_, *d*5-RvD2, *d*4-PGE_2_, and 10 µM *d*8-AA; Cayman Chemical/Biomol GmbH, Hamburg, Germany) to facilitate quantification and sample recovery. Samples were kept at −20 °C for 60 min to allow protein precipitation. After centrifugation (1200× *g*, 4 °C, 10 min), 8 mL of acidified water was added (final pH = 3.5) and the samples were subjected to solid-phase extraction (SPE). The SPE cartridges (Sep-Pak^®^ Vac 6cc 500 mg/6 mL C18; Waters, Milford, MA, USA) were equilibrated with 6 mL of methanol and then with 2 mL of water before sample loading onto the columns. After washing with 6 mL of water and an additional 6 mL of *n*-hexane, LM were eluted with 6 mL of methyl formate. The eluates were brought to dryness using a TurboVap LV evaporation system (Biotage, Uppsala, Sweden) and resuspended in 100 µL of methanol/water (50/50, *v*/*v*) for analysis by UPLC-MS-MS. The LM were analyzed with an Acquity™ UPLC system (Waters, Milford, MA, USA) and a QTRAP 5500 Mass Spectrometer (ABSciex, Darmstadt, Germany), equipped with a Turbo V™ Source and electrospray ionization. LM were separated using an ACQUITY UPLC^®^ BEH C18 column (1.7 µm, 2.1 × 100 mm; Waters, Eschborn, Germany) at 50 °C with a flow rate of 0.3 mL/min and a mobile phase consisting of methanol/water/acetic acid of 42/58/0.01 (*v*/*v*/*v*) that was ramped to 86/14/0.01 (*v*/*v*/*v*) over 12.5 min and then to 98/2/0.01 (*v*/*v*/*v*) for 3 min. The QTrap 5500 was operated in negative ionization mode using scheduled multiple reaction monitoring (MRM) coupled with information-dependent acquisition. The scheduled MRM window was 60 s, optimized LM parameters were adopted, and the curtain gas pressure was set to 35 psi. The retention time and at least six diagnostic ions for each LM were confirmed using external standards (Cayman Chemical/Biomol GmbH, Hamburg, Germany). Where necessary, the identity of low-abundance analytes was confirmed by fragmentation pattern matching after reanalysis on a QTrap 7500 mass spectrometer (Sciex, Framingham, MA, USA) and comparing the enhanced product ion scans of the biological sample with that of authentic standards. As proof of concept of the dynamic modeling of the human intestine, differences in probiotic metabolization of PUFA to biologically significant oxylipins are compared in terms of representative chromatograms instead of presenting the absolute LM amounts.

### 2.10. Data Presentation and Statistical Analysis

Proof of concept of the suitability of the TIM-1/2 models to address our research questions was established by performing two independent experiments in duplicates, which were thus not subjected to further statistical analysis. Data are given as means from two independent experiments performed in duplicates.

## 3. Results

### 3.1. Germination and Survival of Bacillus megaterium DSM 32963 During Gastrointestinal Transit in TIM-1 and TIM-2

#### 3.1.1. Determination of Viable Cells and Spores by Plate Counting

**Figure 3:**
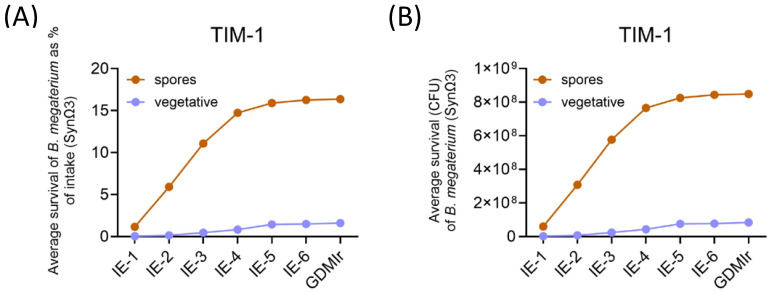
Survival of *Bacillus megaterium* DSM 32963 in TIM-1. Survival expressed as percentage of the initial amount of spores ingested (**A**) and as absolute amount of cells (**B**). The count for vegetative cells = total counts (after direct plating) minus count of spores (after heating to 70 °C). Data are given as means of two independent experiments performed in duplicates. IE: ileal efflux (sampled after 1 h (IE-1) to 6 h (IE-6)); GDMIr: gastric duodenal middle ileal residue (remaining volume of TIM-1 system).

Approximately 35% of the ingested *Bacillus megaterium* DSM 32963 spores were recovered at the end of the TIM-1 system (Figure 3A), corresponding to 8.5 × 10^8^ CFU (Figure 3B). This means that approximately 65% of the input bacteria germinated. Of these germinated cells, most did not survive during transit in the upper GI tract, as only ~3% of the intake was recovered cumulatively at the end of the experiment as germinated cells (Figure 3A).

**Figure 4:**
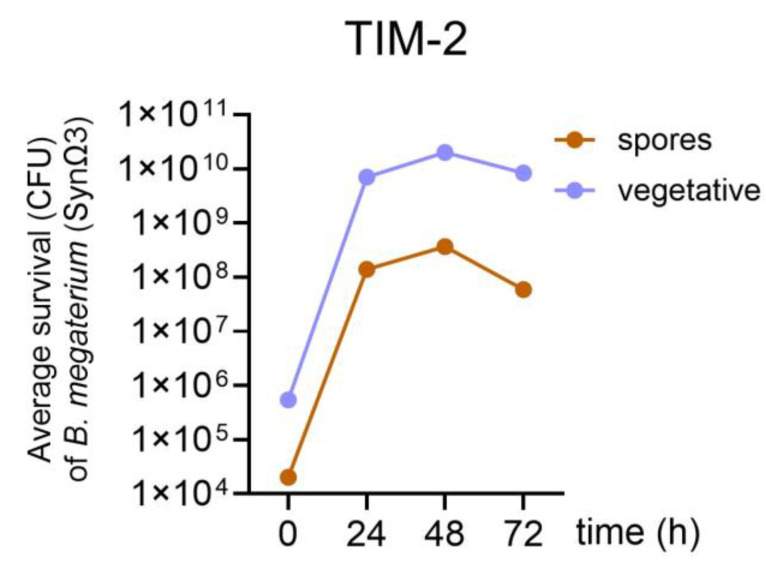
Amounts of vegetative cells and spores—including *Bacillus megaterium* DSM 32963—present in TIM-2. The count for vegetative cells = total counts (after direct plating) minus count of spores (after heating to 70 °C).

Survival and engraftment of *Bacillus megaterium* DSM 32963 in the colon system, TIM-2, was estimated (by plate counting) in experiments with SynΩ3 and under control conditions (SIEM only). Just prior to the addition of the test samples at T0, there were around 10^4^ spores and 10^6^ vegetative cells that could be counted on the chromogenic substrate, indicating a *Bacillus* background population present in the collected fecal donations. However, after addition of the test sample containing the *Bacillus megaterium* DSM 32963 spores, the number of cells detected on the plates increased by approximately 4 log factors (to 10^8^ and 10^10^, respectively, for spores and vegetative cells) at T24 and remained relatively constant after that, with vegetative cells accounting for ~99% of the cells and spores for only ~1% (Figure 4).

#### 3.1.2. Determination of *Bacillus megaterium* DSM 32963 Count by Strain-Specific qPCR

**Figure 5:**
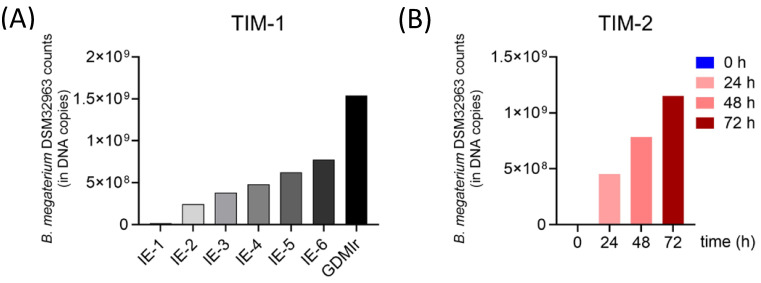
Propagation of *Bacillus megaterium* DSM32963 in TIM-1 and TIM-2. SynΩ-3-supplemented TIM-1 (**A**) and TIM-2 (**B**) systems were assessed at times indicated for *Bacillus megaterium* DSM32963 DNA copy numbers by qPCR using strain-specific primers. Data are given as means of two independent experiments performed in duplicates. IE: ileal efflux (sampled after 1 h (IE-1) to 6 h (IE-6)); GDMIr: gastric duodenal middle ileal residue (remaining volume of TIM-1 system).

We verified the presence of *Bacillus megaterium* DSM 32963 in TIM-1 and TIM-2 by applying a quantitative PCR using strain-specific primers. The specificity of the primer pair was confirmed by the absence of detectable levels of the amplicon in blank-, fish-oil-, and Ω3-salt-supplemented TIM runs. The addition of SynΩ3, though, led to quantifiable amounts of the specific *Bacillus megaterium* DSM 32963 amplicon, which increased over time up to 1.5 × 10^9^ in TIM-1 (Figure 5A). Importantly, we found a similar increase in *Bacillus megaterium* DSM 32963 amplicon numbers in SynΩ3-treated TIM-2 samples (Figure 5B), which reinforces that the expansion of Bacillus sp. (Figure 4) can partly be attributed to an expansion or persistence of *Bacillus megaterium* DSM 32963 on the background of a complex microbiota.

### 3.2. Production of Lipid Mediators During Gastrointestinal Transit in TIM-1 and TIM-2

#### 3.2.1. Production of SPM and Precursors in TIM-1

**Figure 6:**
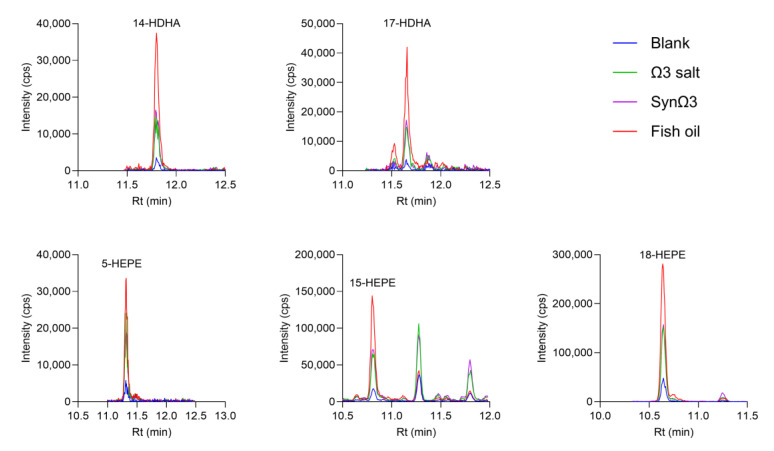
Chromatograms of SPM precursors formed in TIM-1. The indicated treatment groups (blank, Ω3 salt, SynΩ3, and fish oil) were passed through the model comprising the stomach and small intestine and stored at −4 °C until measurement by targeted UPLC-MS/MS. Graphs display representative chromatograms of two independent experiments performed in duplicates.

Several monohydroxylated derivatives of EPA and DHA were detected in the TIM-1 model, including 14- and 17-HDHA, as well as 5-, 15-, and 18-HEPE, whose concentrations were higher in the fish oil group versus the control. The concentrations of these SPM precursors were lower in the Ω3 salt and SynΩ3 group compared to the fish oil group (Figure 6).

#### 3.2.2. Production of SPM and Precursors in TIM-2

**Figure 7:**
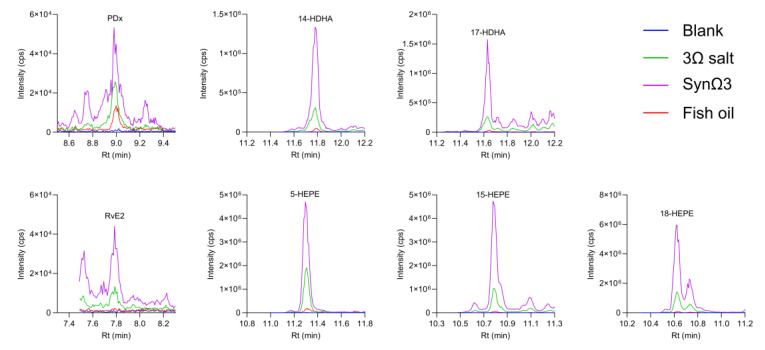
Chromatograms of SPM and SPM precursors formed in TIM-2 after 48 h. The indicated treatment groups (blank, Ω3 salt, SynΩ3, and fish oil) were passed through the model comprising the large intestinal compartments and stored at −4 °C until measurement by targeted UPLC-MS/MS. Graphs display representative chromatograms of two independent experiments performed in duplicates.

In TIM-2, background levels of the “blank” meal were minor or very low for all measured LM (Figure 7).

The *n*-3 PUFA test products had very different effects on the concentrations of SPM as well as mono- and dihydroxylated PUFA. Strongest effects were seen in all cases after inoculation with SynΩ3. For PDX and RvE2, two SPM were formed by SynΩ3; their peak intensities were much higher as compared to the other treatments (Figure 7). In line with this, SPM precursor levels, such as 14-HDHA, 17-HDHA, 5-HEPE, 15-HEPE, and 18-HEPE, were also elevated by SynΩ3 and, to a lesser extent, by the Ω3 salt. Peak intensities of these precursors were two- to ten-fold higher in TIM-2 than those found in fish-oil-treated TIM-1 runs. The differences between SynΩ3 and Ω3 salt groups indicate that the appearance of SPM and SPM precursors in the SynΩ3 group was due to their biosynthesis caused by *Bacillus megaterium* DSM 32963, with only a minor contribution of the microbiota present in TIM-2.

**Figure 8:**
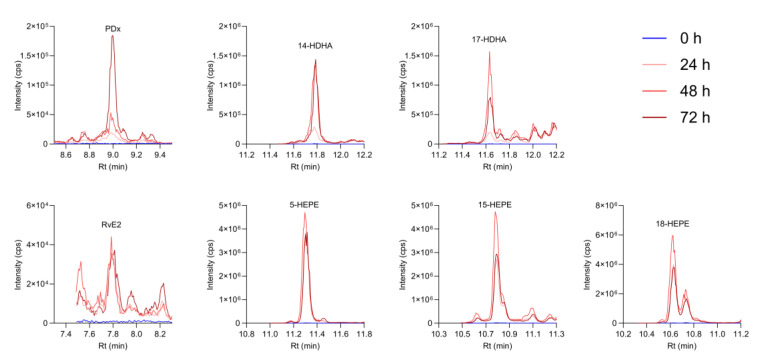
Chromatograms of SPM and SPM precursors formed from SynΩ3 in TIM-2 over time. The model comprising the large intestinal compartments was inoculated with the microbiota on the first day and fed with the SIEM medium during an overnight adaptation period, after which SynΩ3 was added to the model and samples were taken every 24 h. The time point *t* = 0 h is just prior to the addition of SynΩ3. Samples were stored at −4 °C until measurement by targeted UPLC-MS/MS. Graphs display representative chromatograms of two independent experiments performed in duplicates.

Kinetics of SynΩ3-derived SPM and SPM precursors are displayed in Figure 8. PDX levels increased over time, peaking at 72 h, while DHA-derived SPM precursors, such as 14-HDHA and 17-HDHA, peaked at the 48 h time point. RvE2 appeared already after 24 h and then remained at relatively stable amounts throughout the time course. EPA-derived SPM precursors 5-HEPE, 15-HEPE, and 18-HEPE also peaked at 24 h, with slightly decreasing levels at 48 and 72 h.

Control and fish-oil-supplemented experiments did not yield detectable amounts of any of the aforementioned substances. Interestingly, and in contrast to TIM-1, fish oil treatment did not lead to detectable amounts of SPM precursors 14-HDHA, 17-HDHA, or 18-HEPE or their subsequent resolvins or protectins at any time point. This indicates their degradation as well as the fact that they could not be used, or only insufficiently, for de novo generation of SPM.

## 4. Discussion

SPM are potent agonists of inflammation resolution and, therefore, promising candidates to counteract a multitude of human chronic pathologies [25], including IBD [11]. While SPM and related LM can form endogenously from ingested *n*-3 PUFA, this formation is highly variable and can be compromised under certain pathological conditions. This may explain the inconclusive outcomes from fish oil supplementation studies and argues that *n*-3 PUFA availability is not sufficient to achieve inflammation resolution [26,27]. In principle, a dysfunctional SPM production capacity of an organism can be overcome by either direct administration of SPM or by introducing a self-sufficient production system. We aimed for the latter by conceiving a metabiotic approach to target SPM formation/levels in humans, based on the discovery of *Bacillus megaterium* being capable of producing SPM (precursors) from bioavailable *n*-3 PUFA sources in vitro [8].

SynΩ3, a synbiotic *n*-3 PUFA formulation comprising *Bacillus megaterium* DSM 32963 and an *n*-3 PUFA lysine salt, significantly increased plasma SPM precursor levels (e.g., 5-HEPE and 18-HEPE) in a randomized controlled trial, and this effect was stronger than what was achieved by fish oil treatment with a similar *n*-3 PUFA dose [18]. These findings argued for an intraluminal *n*-3 PUFA conversion by *Bacillus megaterium* (DSM 32963) in the GIT, followed by LM absorption into the circulation.

To explore this hypothesis further, we here applied the TIM-1 and TIM-2 models as test systems to answer the following research questions: (i) Is *Bacillus megaterium* DSM 32963 active under simulated, dynamic GIT conditions and in the context of a complex colon microbiota? (ii) Does the LM spectrum formed correlate with what was found in human plasma? (iii) Does the Ω3 salt or a fish oil also act as a substrate for the colon microbiota?

In summary, the synbiotic test product SynΩ3 did not generate LM under simulated gastric and small intestinal conditions—most likely due to poor survival of the applied *Bacillus megaterium* DSM 32963 strain—but very high levels of several SPM precursors (17-HDHA, 5-HEPE, and 18-HEPE) and two SPM (PDX and RvE2) under large intestinal conditions. This pattern aligns well with the induction of plasma 5-HEPE and 18-HEPE in SynΩ3-supplemented humans observed earlier [8].

The Ω3 salt served as an LM-generating substrate for the TIM-2-resident microbiota, albeit LM levels were much higher (up to ~ ten-fold) in the presence of *Bacillus megaterium* DSM 32963. These findings are of clinical relevance as they demonstrate that a gut microbiota—pooled from healthy human donors—is capable of producing LM from *n*-3 PUFA and that this capability is dependent on substrate availability, i.e., intraluminal presence and chemical form of the *n*-3 PUFA (e.g., esterified versus free fatty acids) and oxygen level, as well as the composition and activity of the gut microbiota. Arguably, PUFA-oxygenating microorganisms such as *Bacillus megaterium* may be strong determinants of the biochemical fate of PUFA in the colon. We showed before that many strains of this species, in addition to the strain used here, produce significant amounts of LM and SPM ^8^. As *Bacillus megaterium* sp. has been found in human feces [28] and saliva [29], it is plausible that a human gut microbiota can metabolize *n*-3 PUFA in this direction, as observed here. Other microbial taxa that are likely to contribute as well are *Malassezia* and *Pseudomonas aeruginosa* [30,31,32]. One must consider that fatty acids per se are effectively being absorbed in the small intestine and are consequently not available to bacteria in more distal parts of the GIT. Controlled release formulations, as applied for SynΩ3 [8,18], can, however, enable colon targeting to increase the availability of PUFA to the colon microbiota. At the same time, such a formulation rescues *Bacillus megaterium* DSM 32963 survival by escaping upper GIT conditions. Colon-targeted SynΩ3 and similar formulations are attractive candidates to treat colon-specific inflammatory conditions that are modulated by SPM and SPM precursors, such as Crohn’s disease [12], ulcerative colitis [33], and irritable bowel syndrome [34]. We showed that *Bacillus-megaterium*-induced SPM profiles are highly strain-specific [8], a fact that can be used to tailor synbiotic *n*-3 PUFA compositions based on the intended application or needs for certain LM.

Another interesting finding of our study is that 18-HEPE, 14-HDHA, and 17-HDHA contained in the SPM-precursor-enriched fish oil were found in TIM-1 but disappeared in TIM-2. Presumably, this resulted from their degradation plus lack of de novo synthesis; also, subsequent SPM were not formed in TIM-2 with fish oil. We therefore conclude that SynΩ3 is a more promising strategy to induce intraluminal but also circulating LM levels.

Future studies are warranted to test the clinical effectiveness of SynΩ3 in resolution medicine and the management of inflammatory diseases, such as those mentioned above. Analyzing gut microbiota in *n*-3 PUFA trials may give further insights into possible substrate–microbe interactions that determine host outcomes.

## 5. Conclusions

This study reinforces the concept that gut intraluminal production and subsequent absorption of LM can be triggered by supplementation with SynΩ3. It appears worthwhile to test the clinical effectiveness of SynΩ3 and related approaches that target self-sufficient LM production in situ in chronic inflammatory diseases where SPM are likely to play a beneficial role, e.g., inflammatory bowel diseases and rheumatoid arthritis.

## 6. Patents

WO/2020/109474

WO/2020/109480

## Figures and Tables

**Table 1:** Composition of the meal added to the various TIM-1 experiments.

Blank	SynΩ3	Ω3 Salt	Fish Oil
150 g GES ^1^	150 g GES ^1^	150 g GES ^1^	150 g GES ^1^
150 g LB medium ^2^	150 g LB medium ^2^	150 g LB medium ^2^	150 g LB medium ^2^
	300 mg *n*-3 PUFA lysine salt containing 83.3 mg EPA, 41.7 mg DHA, ~2.4 billion CFU *Bacillus megaterium* DSM 32963 (B4U™63)	300 mg *n*-3 PUFA lysine salt containing 83.3 mg EPA, 41.7 mg DHA	500 mg fish oil containing ~54 mg EPA, ~108 mg DHA
5 mL start residue	5 mL start residue	5 mL start residue	5 mL start residue

^1^ GES: gastric electrolyte solution [22]; ^2^ LB medium: 5 g/L yeast extract, 10 g/L tryptone, 0.5g/L NaCl, and 1 g/L glucose.

## Data Availability

The original contributions presented in this study are included in the article; further inquiries can be directed to the corresponding author.
